# Sun Protection and Tanning Behaviors in Caregivers: Prevalence, Determinants, and Associations with Children’s Behaviors

**DOI:** 10.3390/ijerph19116876

**Published:** 2022-06-04

**Authors:** Katharina Diehl, Karlijn Thoonen, Eckhard W. Breitbart, Annette B. Pfahlberg, Tatiana Görig

**Affiliations:** 1 Department of Medical Informatics, Biometry and Epidemiology, Friedrich-Alexander-Universität Erlangen-Nürnberg (FAU), 91054 Erlangen, Germany; annette.pfahlberg@fau.de (A.B.P.); tatiana.goerig@fau.de (T.G.); 2 Centre for Environmental Safety and Security, National Institute for Public Health and the Environment, 3721 MA Bilthoven, The Netherlands; karlijn.thoonen@rivm.nl; 3 Arbeitsgemeinschaft Dermatologische Prävention, 21614 Buxtehude, Germany; info@professor-breitbart.de

**Keywords:** sun protection, sun safety, children, caregivers, sunburn, tanning, Germany

## Abstract

The association between skin cancer and ultraviolet radiation (UVR) is well established, and sun protection behavior represents an important preventative measure. In children, caregivers play a key role in this regard. The subject of this study was threefold: whether caregivers of 1 to 11 year-old children are more likely to use sun protection measures compared to non-caregivers, whether considering oneself a role model is associated with sun protection behaviors, and whether their sun protection and risk behaviors are related to children’s behaviors. We used data from the 2020 wave of the National Cancer Aid Monitoring (NCAM) comprised of 4000 individuals (including 554 caregivers of at least one child aged 1–10 years) aged 16 to 65 years and living in Germany. Data were collected through telephone interviews between October and December 2020. No significant differences between caregivers and non-caregivers regarding sun protection and risk behaviors were identified (except tanning on vacation). In both groups, sun protection behaviors were deficient. Caregivers who considered themselves role models concerning sun safety were more likely to use sun protection measures (e.g., using sunscreen on the face: OR = 5.08, *p* < 0.001). In addition, caregivers’ sun protection behaviors were positively associated with children’s behaviors. Caregivers being highly protected against UVR were more likely to report the use of different measures by/in the child (mean = 4.03), compared to caregivers with medium (3.41) and low (2.97, *p* < 0.001) protection levels. However, we also found that caregivers’ risk behavior was associated with children’s reported risk behavior. For future prevention, it might be worth focusing on the aspect of caregivers serving as role models. A comprehensive public-health strategy is needed, including key figures such as pediatricians to prevent today’s children from developing skin cancer in later life.

## 1. Introduction

Skin cancer—including malignant melanoma and keratinocyte carcinoma—is the most common form of cancer worldwide [1]. Despite its essential role for normal growth and skeletal development [2], ultraviolet (UV) radiation remains one major risk factor for the development of skin cancer [3]. To reduce skin cancer morbidity and mortality, proper sun protection should start early in life [4]. This seems especially important since a relatively higher cumulative amount of UV radiation is acquired in childhood and early adolescence compared to adulthood [5,6,7], and unprotected UV-exposure and sunburn in this phase is associated with increased risk of all skin-cancer types [5,8,9].

Up to now, many studies from all over the world have focused on sun protection in children. Especially in early childhood, parents play a key role in children’s sun safety [10,11,12]. First, parents are responsible for their children’s sun safety, especially when they are young and unable to perform their own sun protection behaviors yet [13]. Second, parents function as important role models for their children and, through their own sun protection behavior, can shape their children’s protection behavior and their attitudes toward sun protection in later life [14,15]. An important aspect may be the self-efficacy expectations of parents that were shown to be positively associated with parental sun protection for the child [16,17].

Although parents play a vital role in children’s sun safety, studies concerning their own behaviors demonstrate inconsistent findings. A French study, for example, showed that both adults with and without children did not significantly differ in their sun protection measures (except regarding their usage of sunscreen with a higher SPF [18]). However, parents who consider themselves role models and set a good example seem to be more likely to use sun protection measures for their children [11,19]. As found in other studies, parents who practiced sun protection behaviors for themselves were more likely to report performing sun protection measures for their children [20,21,22,23,24]. However, several studies have shown that parents were more likely to focus on sun protection practices for their children than for themselves [18,25,26].

Some of the aforementioned associations are understudied or focused on specific groups (e.g., parents whose relative was diagnosed with melanoma [20]). In addition, while previous research indicates that both parents and people in the general population often have a positive attitude regarding tanned skin [19,27,28], little is known about whether sun protection and tanning behaviors differ between caregivers and non-caregivers. Furthermore, the question of whether parents who consider themselves as role models for sun protection also act like role models remains open. To further investigate the association of parental sun protection as well as tanning behavior and their children’s behavior, a nationwide study is imperative.

Based on the current stage of research, the aim of our study was to answer the following four research questions on caregivers’ own sun protection and tanning behaviors as well as the associations of caregivers’ sun protection and tanning behaviors with children’s behaviors by using a nationwide sample from Germany:

1. Do caregivers of young children differ in the sun protection and tanning behaviors from non-caregivers?
2. Are caregivers who perceive themselves as role models for sun protection more likely to use sun protection measures for themselves compared to caregivers who do not consider themselves role models?
3. Is a caregiver’s use of sun protection measures associated with the same use in the child, including the use for the child as well as the child’s own behavior?
4. Are caregivers’ tanning behaviors and frequency of sunburn associated with children’s tanning behavior and sunburn, respectively?

## 2. Materials and Methods

### 2.1. Study Setting

Study data were collected within the sixth cross-sectional wave of the National Cancer Aid Monitoring [29]. From October to December 2020, 4000 individuals aged 16–65 years living in Germany were questioned in computer-assisted telephone interviews (response rate: 29.8%). Participants were selected via a two-stage random sampling procedure: first, the household was contacted on a random basis; and second, the interview partner was identified at the household level by using the next-birthday-method [30]. All participants provided verbal informed consent to participate in the study. The Ethics Committee II of the Medical Faculty Mannheim, Heidelberg University, approved the study (2017-662N-MA).

This manuscript is based on the data of 4000 participants, that include a sub-sample of 554 caregivers. Caregivers were identified based on the self-report to live in the same household as one or more children aged between 1 and 10 years [11]; these parameters correspond to suggestions in previous research [22]. In households with more than one child of this age, we asked caregivers to shape their responses according to the oldest child in this age group, as suggested in previous studies [12,31,32,33].

### 2.2. Instrument and Measures

All questions and response categories from the questionnaire were intensively pretested in fifteen cognitive interviews, with a focus on the comprehensibility of questions and response categories as well as the difficulty in providing answers.

#### 2.2.1. Variables Assessed for Caregivers and Non-Caregivers

*Sociodemographic characteristics*: The participants provided information on their sex (male vs. female), age, immigrant background (resulting in the categories yes vs. no, based on [34]), the highest level of school education (categorized as low [still at school, without school-leaving qualification or general school], medium [secondary school, i.e., 10 years of school education], or high [high school graduate, i.e., 12–13 years of school education]), and employment status (categorized as unemployed,. part-time employed, or full-time employed).

*Skin type:* We collected information on the self-reported skin type of the participants using Fitzpatrick’s classification [35].

*Sun protection behaviors:* The following sun protection behaviors were assessed: use of sunscreen or day cream with sun protection factor on the face, use of sunscreen on the body, wearing a shirt with sleeves that covers the shoulders, wearing headgear, using sunglasses, seeking shade (e.g., under an umbrella). The respondents were asked to estimate their behaviors when being outside on a sunny summer day for at least 10 min [31,36,37]. For the analyses, the responses were dichotomized (always, often vs. sometimes, rarely, never).

In addition, for comparison of sun protection behaviors in caregivers and in children, a score was calculated. Thus, the six dummy variables (one variable each for the respective behaviors) were summed up (values 0–6) and grouped into three categories: low use (0–1 measures used always or often), medium use (2–4 measures), and high use (5–6 measures) of sun protection.

*Tanning behaviors:* One-year-prevalence of tanning bed use (yes or no) was assessed [29]. Additionally, intentional outdoor tanning on three different occasions was assessed: “How often do you go in the sun in order to get a tan during your holidays?”; “In summer, how often do you go in the sun in order to get a tan on the weekends?”; and “In summer, how often do you go in the sun in order to get a tan on a typical workday?” [11]. Response categories were dichotomized into (1) very often, often, and sometimes, and (2) rarely and never. To assess sunburn in the last 12 months, we used the validated question from Glanz et al. [31]: “In the past 12 months, how many times did you have a red or painful sunburn that lasted a day or more?”. We dichotomized responses as follows: never vs. once or more often.

*Role modelling:* Participants’ agreement about being a role model regarding sun protection for their children was assessed with the statement “I try to set a good example with my own sun protection” [22]. The response categories were dichotomized (strongly disagree, rather disagree vs. rather agree, strongly agree).

*Risk perceptions regarding UV-radiation*: Risk perception was assessed with three items: “Every sunburn leaves permanent damage in the skin”, “Regular use of solariums causes the skin to age prematurely”, and “Regular tanning bed use increases the risk of skin cancer” (rather agree vs. rather not agree).

#### 2.2.2. Variables Assessed for Children

Caregivers provided proxy reports on the application of recommended sun protection measures by answering the following questions: “How often is sunscreen applied on the child’s face?”; “How often is sunscreen applied on other body areas exposed to the sun?”; “How often does the child wear a shirt with sleeves that cover the shoulders?”; “How often does the child wear a cap or a hat?”; “How often does the child wear sunglasses?”; and “How often does the child stay in the shade or under an umbrella?” The participants were asked to estimate how often these measures were used on a sunny summer day when the child was outside for at least ten minutes [11,31]. The response categories were dichotomized (always, often vs. sometimes, rarely, never). For additional comparison of behaviors in caregivers and in children, a sum score of the six behavioral dummy variables was calculated ranging from 0 to 6 that was used as a metric variable.

The 12-month prevalence of children’s sunburn was assessed with the question “In the past 12 months, how many times did this child have a painful or red sunburn that lasted a day or more?”, as recommended by Glanz et al. [31]. The response categories were dichotomized (never vs. once or more often). In addition, we included the question “How often does the child spend time in the sun in order to get a tan?” [31] and dichotomized the responses (never, rarely, sometimes vs. often, very often).

### 2.3. Statistical Analyses

Our analysis followed the four research questions stated in the introduction:

(1) To explore whether caregivers and non-caregivers differed regarding their sociodemographic characteristics, sun protection behaviors, and tanning behaviors, we used chi-squared tests for categorial variables and Mann-Whitney-U tests for quantitative variables. For use of sun protection measures and tanning behaviors, additional logistic regression analyses adjusted by sex, age, immigrant background, education level, employment, and skin type were performed.
(2) For analyzing potential differences in sun protection behaviors based on whether caregivers considered themselves a role-model, chi-squared tests were calculated. For significant associations, additional logistic regressions were performed, and these were adjusted for sex, age, immigrant background, education level, employment, and skin type.
(3) To analyze potential associations between the sun protection behaviors of caregivers and of children, chi-squared tests were performed. In addition, Kruskal-Wallis-test was used to analyze the association of the sun protection score of children with the caregivers’ use of sun protection grouped into three categories. Here, mean values and standard deviations (SD) are reported.
  Additional linear regression models were calculated to control for potential confounding. First, a crude model was calculated that included the number of sun protection measures in the child (dependent variable) and the caregiver (independent variable). Then, a model adjusted for caregivers’ sociodemographic characteristics and skin type as well as a model adjusted for children’s age, sex, and skin type were performed.
(4) In addition, chi-squared tests were used to identify associations between caregivers’ and children’s risk behaviors (i.e., sunburn and tanning behavior). Fisher’s exact test was performed for tanning behavior, because case numbers were low. For sunburn, additional adjusted logistic regression analysis was calculated.

All analyses were performed with SPSS version 27 (IBM Corporation, Armonk, NY, USA) with a predefined level of significance of *p* < 0.05.

## 3. Results

About half of included caregivers were female, mean age was 34.5 (SD: 11.4) years (Table 1). More than half of the caregivers (58.5%) had a higher education level and nearly nine out of ten were full-time or part-time employed. Caregivers were significantly younger than non-caregivers (*p* < 0.001), were more likely to have a higher education level (*p* = 0.007), and were employed in a greater proportion (*p* < 0.001; Table 1).

### 3.1. Comparison of Caregivers and Non-Caregivers

Caregivers and non-caregivers differed significantly with regard to their use of sunscreen, their tanning behaviors, and sunburn occurrence in bivariate analysis (Table 2). However, when controlled for sex, age, immigrant background, education level, employment, and skin type, only the difference in outdoor tanning during vacation remained significant: caregivers were more likely to tan (very) often outdoors during vacation compared to non-caregivers (OR = 1.45 [1.13–1.86], *p* = 0.003). No significant differences were found for risk perceptions regarding UV-radiation (not shown).

### 3.2. Role Modelling

To answer the second research question, we compared sun protection behaviors of caregivers who considered themselves role models for their children regarding sun protection with those of caregivers who did not indicate that they considered themselves role models. We found that caregivers who considered themselves role models were more likely to use sunscreen on their face (48.7% vs. 17.4%, *p* < 0.001), on their body (45.7% vs. 10.5%, *p* < 0.001), and to seek shade (very) often (71.7% vs. 53.5%, *p* = 0.001). No association was found for wearing headgear, sunglasses, and a shirt that covers the shoulders. Significant findings were confirmed in adjusted logistic regression analysis for sunscreen use on the face (OR = 5.08 [2.57–10.05], *p* < 0.001) and on the body (OR = 7.22 [3.28–15.90], *p* < 0.001) as well as for seeking shade (very) often (OR = 2.27 [1.33–3.87], *p* = 0.003).

### 3.3. Association of Protection Behaviors in Caregivers and Children

**Figure 1:**
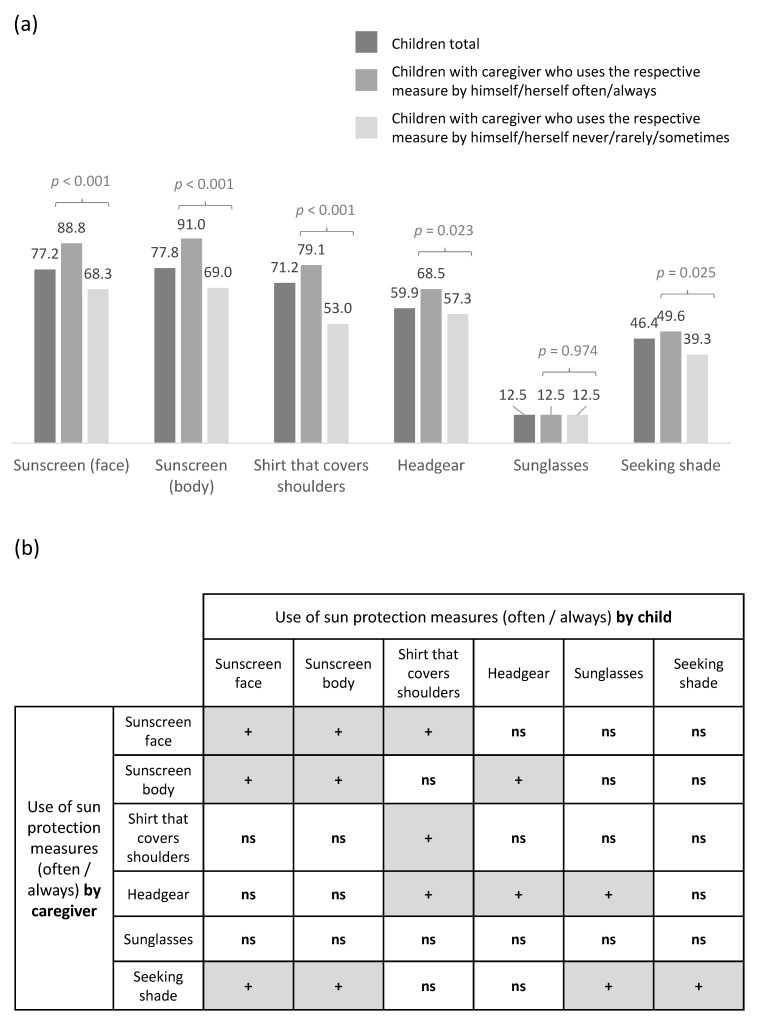
Associations of sun protection behaviors in caregivers and children. (**a**) Measures often/always used by the child stratified by the caregiver’s use. (**b**) Associations of measures used by the caregiver and those used by the child. +: chi-squared test was significant on *p* < 0.05 level of significance; ns: not significant association.

Caregivers’ sun protection behaviors were significantly associated with sun protection behaviors in their child: Caregivers who used sunscreen on their face and body often/always wore shirts that covered their shoulders and headgear often/always, and those who sought shade often/always were more likely to report the same sun protection behaviors for their child (Figure 1a). Figure 1b shows that some sun protection behaviors were interrelated.

**Figure 2:**
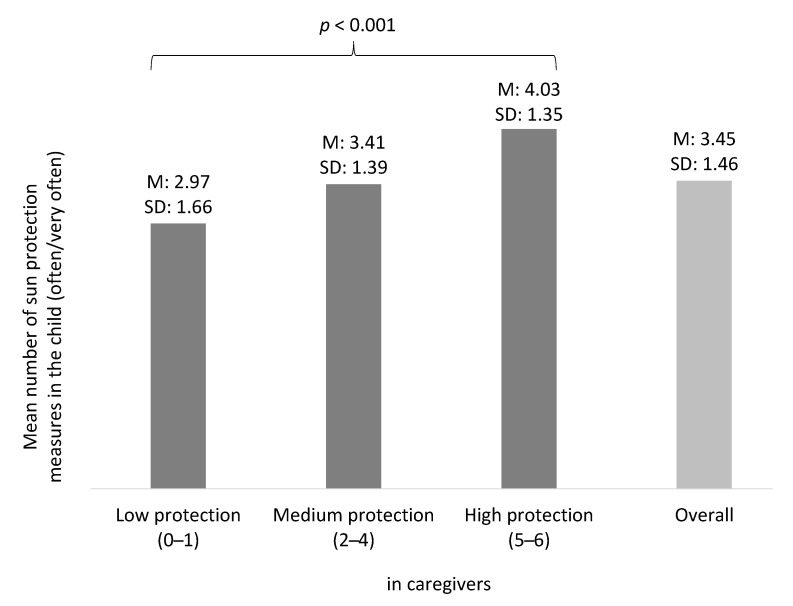
Mean number of sun protection measures used in the child according to caregivers’ protection status. *p*-value based on Kruskal-Wallis-test; M: Mean; SD: Standard deviation; reported is the mean value of children’s sun protection measures by sun protection measures in caregivers (low vs. medium vs. high protection) Mean of measures used by the caregivers: 2.99 (SD: 1.47).

In addition to investigating individual sun protection behaviors, we calculated a score of the six behaviors based on the dummy variables presented in Figure 1. We found that caregivers who protect themselves at a high level are more likely to report a higher number of measures used in/by the child (*p* < 0.001; Figure 2).

To control for potential confounding, we conducted linear regression analysis with the number of sun protection measures in the child as a dependent variable. The association of sun protection measures in the child and in the caregiver (used as metric variable; β_standardized_ = 2.685, *p* < 0.001) remained stable after controlling for children’s (β_standardized_ = 0.241, *p* < 0.001) and caregivers’ sociodemographic and skin characteristics (β_standardized_ = 0.230, *p* < 0.001).

### 3.4. Association of Risk Behaviors of Caregivers and Children

Besides sun protection behaviors, we analyzed whether caregivers’ frequency of sunburn was associated with children’s sunburn and whether the tanning behavior of caregivers was related to children’s tanning behavior. We found that caregivers who experienced sunburn at least once during the previous 12 months were more likely to report sunburn in their child during the last 12 months (31.2%), compared to caregivers who did not experience sunburn (16.2%, *p* < 0.001). This finding was confirmed in logistic regression analysis adjusted for sociodemographic variables and caregivers’ skin type (OR = 2.39, *p* < 0.001).

In addition, we found significant associations for tanning behavior, although the number of children who intentionally tan outdoors (very) often was small (3.1%, n = 17). Caregivers who intentionally tanned outdoors (very) often on weekends were more likely to report that their child tans outdoors (very) often compared to those caregivers who tan outdoors on weekends less frequently (7.0% vs. 1.7%, *p* = 0.003). Similar findings were identified for caregivers who tanned outdoors (very) often on weekdays (7.4% vs. 1.9%, *p* = 0.004). Due to the low case number, no adjusted analyses were calculated for this aspect.

## 4. Discussion

Our nationwide findings integrate well with and extend the current state of research. The results described underline that both sun protection behavior and tanning behavior of caregivers are associated with their children’s respective behaviors. We found that the self-perception of a role model for the child among caregivers was an important aspect for sun protection behavior as well. Both findings seem to be a valuable starting point for future prevention measures.

### 4.1. Comparison of Caregivers and Non-Caregivers

We found that caregivers did not differ in their sun protection behaviors from non-caregivers, while illustrating deficits in both groups [38]. A French study comparing parents with non-parents could not identify differences in sun protection behaviors between both groups, but found that the use of sun protection measures was adequate [18]. Although the findings of our study and the French study may not be directly comparable due to differences in the definition of groups, our finding is an indication that, despite their role perception, caregivers of young children do not protect themselves from the sun any better than the general population does. This is worrying, since a lack of sun protection in caregivers could have a negative impact on children’s sun safety. Here, enhancing self-efficacy expectations of use of sun protection measures may help to increase use in adults and may also positively influence use in children [16]. In addition, we found that caregivers of 1 to 10 year-old children were more likely to tan outdoors during vacation. This may be due to the fact of different vacation styles between individuals with younger children, individuals with older children, and those without children.

### 4.2. Role Modelling

In general, the use of sun protection measures among caregivers was low: wearing a shirt that covers the shoulders was often or always performed by 70%, while wearing headgear was the least used measure (23%). This finding is in accordance with the rather low use of sun protection in the general population of Germany [38], and other countries; e.g., see [39,40].

Caregivers who consider themselves role models regarding sun safety were more likely to use sunscreen and to seek shade. This means that these caregivers do not only consider themselves role models but are also more likely to act as role models. In previous analyses, we revealed that caregivers’ self-perception as a role model is also associated with use of sun protection measures in the child [11]. This supports the assumption that the caregivers’ attitude as well as their own sun protection behaviors are important for children’s behavior [21,41,42,43] and deserve particular attention in future prevention campaigns [11].

### 4.3. Association of Protection Behaviors in Caregivers and Children

We found that caregivers’ use of sun protection-measures is associated with children’s use. This applies for single sun protection measures and for the use of different measures at the same time: Well-protected caregivers are more likely to have a well-protected child. Some previous studies, which were performed outside Europe, showed similar associations [20,21,22].

Nonetheless, it should be mentioned that not only the behavior of caregivers is important but also settings such as the kindergarten play an important role for sun protection in childhood [44,45]. Caregivers are able to train and sensitize the child for the use of sun protection measures, but contextual conditions in other settings, such as school and kindergarten, must also be conducive to its use in order for it to be implemented.

### 4.4. Association of Risk Behaviors of Caregivers and Children

At the same time, we found that caregivers who experienced sunburn during the last 12 months were more likely to report sunburn in their child. This may indicate that sun protection measures are probably not appropriately applied, which can result in an unhealthy behavioral pattern among children as well. Previous studies in adult populations show that the use of sunscreen may give a false sense of security from sunburn [46,47] and tempt people to stay longer in the sun, which can result in sunburn. This overreliance on an inadequate use of sunscreen, increasing the risk of sunburn, were also seen among parents in the Netherlands [19]. Caregivers should be made aware that even with sun protection measures there is no absolute protection against UV radiation, and the best protective strategy is to reduce the time spent outdoors during the UV-intensive hours around midday.

However, it may also be the case that some caregivers may not want to admit that the child was sunburnt. Social desirability may play a role here. Such a response behavior may strengthen the statistical association of caregivers’ and children’s sunburn. To explore this aspect and to describe the extent to which caregivers perceive a child’s sunburn as a personal failure, the use of qualitative research (e.g., direct observation [48,49]) may be helpful.

Among the caregivers that intentionally tanned outdoors, similar behavior for their children was also reported. This implies that children not only learn from their caregivers about how to protect themselves from the sun, but also about tanning ideals and behaviors that are harmful for the skin. This aspect should be further investigated, since in our sample the number of children who intentionally tanned was (fortunately) very small.

### 4.5. Implications

Our findings provide important directions for future preventative efforts. Healthcare providers should be sensitized to the topic of sun protection and tanning behavior in children. Pediatricians, who have contact with both children and parents, can play an important role [50]. Previous research showed that pediatricians are aware of the important role of skin cancer prevention [50]. As part of regular health check-ups, they can discuss the benefits of appropriate sun protection not only for the child but also for the skin health of the parents themselves, which can have a positive impact on sun-safety behavior [51]. Additionally, pediatricians can inform caregivers about the health risks of sunbathing. The authors suggested motivational interviewing as a possible method that may lead to higher sun-safety behavior [52]. In addition, pediatricians need to be supported by a broader public-health approach [53]. The same applies to teachers and staff in nurseries and kindergartens. Here, educational campaigns including the use of mobile applications might be helpful.

However, our data suggest that sun protection behaviors in both caregivers and non-caregivers of young children could be improved alongside sun protection behaviors in children. This implies that counselling by health professionals is not only needed with regard to children but also for adults. In addition, knowledge should be improved by educational campaigns targeting parents and the general population.

### 4.6. Study Limitations

A limitation that should be discussed is the use of caregivers’ proxy reports to describe sun protection and tanning behaviors in children. The provided information may be biased due to difficulties in recalling behaviors and due to social desirability. However, proxy reports have widely been used in previous studies [22,32] and seem to be the only alternative to direct observation, because children in the studied age group may not be able to answer a questionnaire on their own. In addition, our study does not provide information on the duration of sun exposure during UV-intensive peak hours between 10 am and 4 pm; nor did we assess days of chronic sun exposure. Moreover, we cannot conclude that the caregivers interviewed in our study are the parents of the children, because we refrained from asking for this sensitive information [11]. Nonetheless, this method was already established in previous nationwide surveys [22], and it can be assumed that adults who live in the same household with a child are relatively well informed about the children’s tanning and sun-safety behavior [11]. In our study, we focused only on sun protection behaviors during summer, although sun protection might be indicated in every season. Thus, sun-protective practices of caregivers—e.g., in winter—is an aspect that still needs to be explored in future research. An additional weakness may be that our sample included a large proportion of caregivers with higher school education. Further, since our study included several sun-protective behaviors in the caregiver and the child, we were not able to examine in detail, for example, sun related knowledge of caregivers or whether sunscreen use is in line with national and international recommendations (e.g., regarding time of application and re-application). These aspects should be studied in future research projects. In addition, to handle the variety on data, we dichotomized several variables, which may limit the findings. 

## 5. Conclusions

Our nationwide data show deficits in the sun protection behaviors of caregivers. However, caregivers who protect themselves often or always are more likely to report more comprehensive sun protection for the children under their care. In addition to that, caregivers who consider themselves role models regarding sun protection are also more likely to indicate that the child uses sun protection measures. Therefore, the aspect of role models should play a major role in education and prevention. Key figures in the field of prevention, such as pediatricians, could emphasize the importance of caregivers being a role model with regard to sun protection. In addition, preventative campaigns should inform the public about the short-term effects of inadequate sun protection behaviors use such as sunburn, but also about long-term effects such as an increased risk for developing skin cancer in later life.

## Figures and Tables

**Table 1:** Sociodemographic characteristics of caregivers compared to non-caregivers.

Characteristic (*n*)	Caregivers % (*n*)	Non-Caregivers % (*n*)	*p*-Value
Sex (3997)			0.203
Male	46.0 (255)	48.9 (1685)	
Female	54.0 (299)	51.1 (1758)	
Age (3913)			**<0.001**
Mean	**34.5 (SD:11.4)**	**46.5 (SD:12.0)**	
Immigrant background (3987)			0.687
No	85.2 (472)	85.8 (2947)	
Yes	14.8 (82)	14.2 (486)	
Education level (3504)			**0.007**
Low	**15.8 (74)**	**16.2 (493)**	
Medium	**25.6 (120)**	**32.4 (984)**	
High	**58.5 (274)**	**51.4 (1559)**	
Employment (3843)			**<0.001**
None	**12.1 (64)**	**23.2 (770)**	
Part-time	**29.4 (155)**	**20.7 (687)**	
Full-time	**58.4 (308)**	**56.1 (1859)**	
Skin type (3976)			0.355
I/II	38.4 (212)	36.4 (1245)	
III-VI	61.6 (340)	63.6 (2179)	

Significant associations are displayed in bold font. Caregivers were individuals living in the same household with at least one child aged 1 to 10 years; Non-caregivers were not living in the same household with children aged 1 to 10 years.

**Table 2:** Sun protection and tanning behaviors in caregivers compared to non-caregivers.

Characteristic (*n*)	Caregivers % (*n*)	Non- Caregivers % (*n*)	*p*-Value	OR ^a^ [95% CI] Caregivers	*p*-Value
Use of sun protection measures					
Use of sunscreen on the face (3996)			0.460		0.875
Often/always	43.7 (242)	42.0 (1446)		1.02 [0.82–1.27]	
Never/rarely/sometimes	56.3 (312)	58.0 (1996)		Ref.	
Use of sunscreen on the body (3996)			**0.001**		0.308
Often/always	**40.1 (222)**	**32.8 (1128)**		1.12 [0.90–1.40]	
Never/rarely/sometimes	**59.9 (332)**	**67.2 (2314)**		Ref.	
Wearing a shirt that covers shoulders (3993)			0.090		0.109
Often/always	70.0 (388)	73.5 (2527)		0.83 [0.65–0.93]	
Never/rarely/sometimes	30.0 (166)	26.5 (912)		Ref.	
Wearing headgear (3996)			0.271		0.325
Often/always	22.9 (127)	25.1 (864)		0.88 [0.68–1.14]	
Never/rarely/sometimes	77.1 (427)	74.9 (2578)		Ref.	
Wearing sunglasses (3997)			0.182		0.803
Often/always	54.0 (299)	50.9 (1753)		0.97 [0.78–1.21]	
Never/rarely/sometimes	46.0 (255)	49.1 (1690)		Ref.	
Seeking shade (3990)			0.217		0.969
Often/always	68.4 (378)	70.9 (2438)		1.00 [0.79–1.25]	
Never/rarely/sometimes	31.6 (175)	29.1 (999)		Ref.	
Tanning behavior					
Current use of tanning beds (3997)			0.134		0.362
Yes	4.3 (24)	5.9 (204)		0.80 [0.49–1.29]	
No	95.7 (530)	94.1 (3,239)		Ref.	
Outdoor tanning on vacation (3996)			**<0.001**		**0.003**
Very often or often	**27.4 (152)**	**19.4 (667)**		**1.45** **[1.13–1.86]**	
Never to occasionally	**72.6 (402)**	**80.6 (2775)**		**Ref.**	
Outdoor tanning at the weekend (3997)			**<0.001**		0.193
Very often or often	**25.6 (142)**	**19.1 (685)**		1.18 [0.92–1.53]	
Never to occasionally	**74.4 (412)**	**80.9 (2785)**		Ref.	
Outdoor tanning on weekdays (3993)			**0.001**		0.241
Very often or often	**22.0 (122)**	**16.2 (558)**		1.17 [0.90–1.53]	
Never to occasionally	**78.0 (432)**	**83.8 (2881)**		Ref.	
Sunburn in the last 12 months (3987)			**0.001**		0.270
At least once	**36.2 (200)**	**28.9 (994)**		1.14 [0.91–1.43]	
Never	**63.8 (353)**	**71.1 (2440)**		Ref.	

^a^ adjusted for sex, age, immigrant background, education level, employment, and skin type; OR: odds ratio; CI: confidence interval; Ref.: reference category; Significant associations are displayed in bold font. Caregivers were individuals living in the same household with at least one child aged 1 to 10 years; Non-caregivers were not living in the same household with children aged 1 to 10 years.

## Data Availability

The data presented in this study are available upon reasonable request from the corresponding author.
